# New insights in swine model of ventricular tachycardia using quantitative myocardial tissue characterization

**DOI:** 10.1186/1532-429X-17-S1-Q132

**Published:** 2015-02-03

**Authors:** Sébastien Roujol, Tamer A Basha, Cory M Tschabrunn, Kraig V Kissinger, Mark E Josephson, Warren J Manning, Elad Anter, Reza Nezafat

**Affiliations:** 1 Department of Medicine, BIDMC / Harvard Medical School, Boston, MA, USA; 2 Radiology, BIDMC / Harvard Medical School, Boston, MA, USA

## Background

Ventricular tachycardia (VT) is often responsible for sudden cardiac death and is generally triggers by the presence of reentry circuits related to a chronic myocardial scar. We have recently developed a novel swine model of VT, where sustained monomorphic reentrant VT can be induced in all animals. This new model offers exciting opportunities for better understanding the underlying substrate of VT, as well as for the development of new mapping and ablation strategies. In this study, we sought to provide in-vivo tissue characterization of this model using myocardial tissue characterization techniques of T_1_, T_2_ and high-resolution LGE.

## Methods

A novel swine model of reentrant VT was induced in 11 Yorkshire swine by 180 min balloon occlusion of the mid left anterior coronary artery. Each animal underwent an in-vivo CMR exam using a 1.5 T Philips scanner at 52±13 days after infarction, followed by an electrophysiology study with programmed stimulation to assess for VT inducibility. During imaging, each animal was sedated, intubated and mechanically ventilated. Native T_1_ mapping using MOLLI (1) and T_2_ mapping (2) were performed and followed by bolus injection of 0.2 mmol/kg of gadobenate dimeglumine and post-contrast T_1_ mapping using MOLLI. All these parametric sequences used ECG-triggered single shot acquisitions with balanced-SSFP imaging readout and the following parameters: (TR/TE=4.3/2.1ms, flip angle=35°(T_1_ mapping)/85°(T_2_ mapping), FOV=360×276 mm^2^, voxel size=2×2 mm^2^, slice thickness=8 mm, 10 slices (T_1_ mapping)/5 slices(T_2_ mapping), SENSE factor=2). Finally, high resolution LGE (3) was performed using a free breathing navigator-gated inversion recovery gradient echo sequence with the following parameters (TR/TE/α=6.5/3.0ms/25˚, FOV=270×270×112 mm^3^, voxel size=1×1×1 mm^3^, compressed sensing factor=4). All imaging was performed in the short axis orientation. Analysis was performed offline using an in-house platform. The areas of enhancement in LGE data was used to visually guide a manual segmentation of the corresponding areas in all T_1_ and T_2_ maps. A similar approach was used to delineate an area of healthy myocardium all T_1_ and T_2_ maps. T_1_/T_2_ maps with artifacts were discarded from the analysis. Native T_1_ times and T_2_ times are reported for both "remote area" and "area of enhancement".

## Results

**Figure 1:**
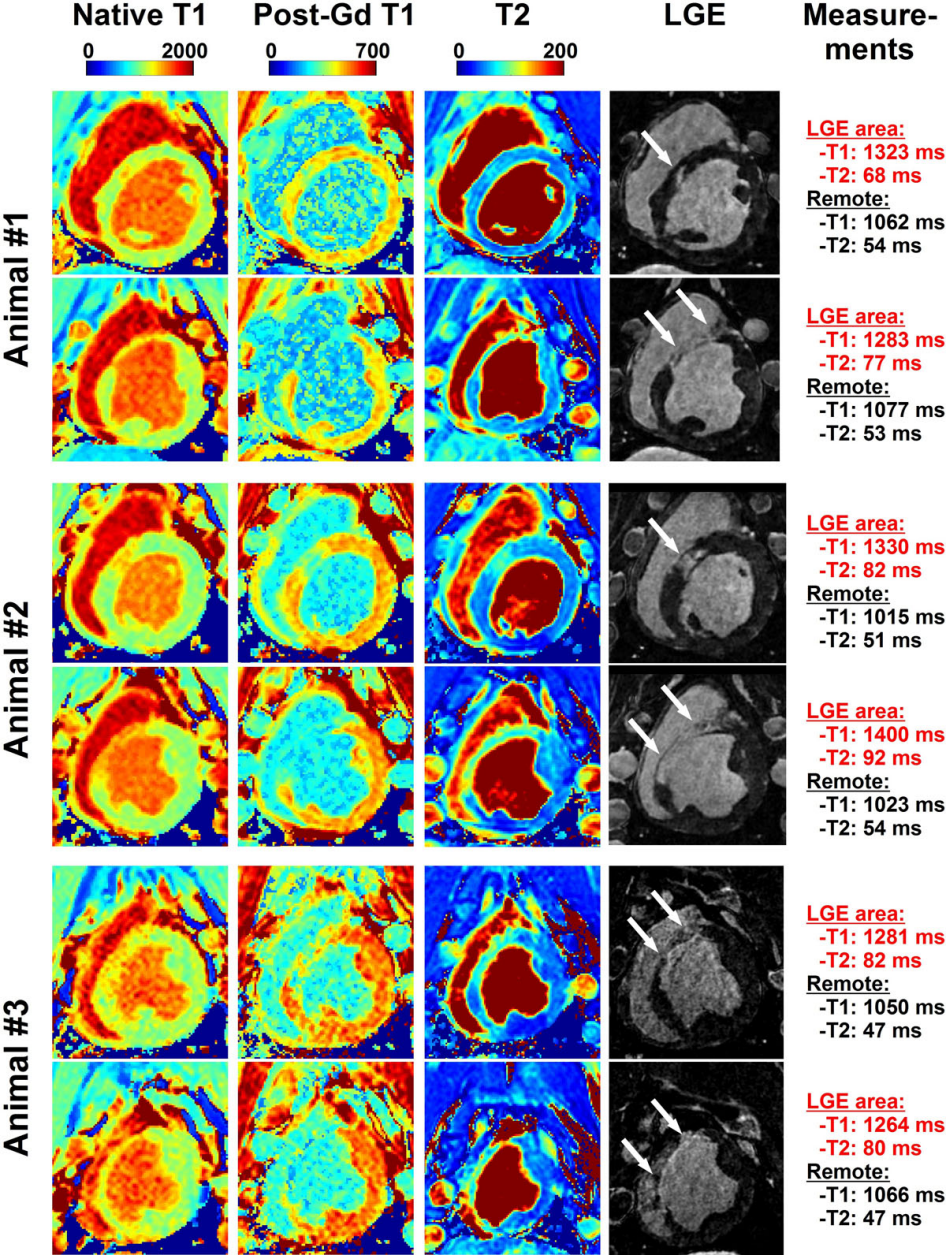
Example of native T_1_ maps, post-contrast T_1_ maps (post-Gd T_1_), T_2_ maps and LGE obtained in 3 swine. Elevated native T_1_ times and T_2_ times can be observed in the area of LGE enhancement.

**Figure 2:**
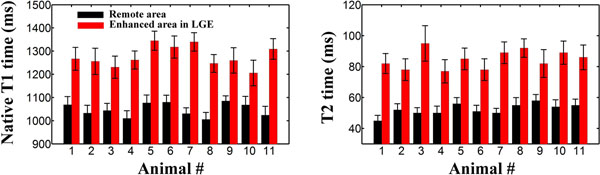
Native T_1_ times and T_2_ times measured in area of enhancement in LGE (red) and in remote area (black). Higher T_1_ times and T_2_ times were obtained in area of enhancement in LGE (p<0.001).

Sustained reentrant VT could be induced in all animals. In-vivo CMR revealed that areas with elevated native T_1_ times and T_2_ times were in good agreement with areas depicting reduced post-contrast T_1_ times and enhancement in LGE (Figure 1). Over all animals, area with enhancement as defined by LGE had higher native T_1_ times (1276±45 vs. 1047±29, p<0.001) and higher T_2_ times (85±6 vs. 52±4, p<0.001) than remote area (Figure 2).

## Conclusions

In this swine model of reentrant VT, areas of LGE hyperenhancement are associated with elevated native T_1_ times and T_2_ times.

## References

[B1] Messroghli MRM 2004

[B2] Akçakaya MRM 2014

[B3] Akçakaya Radiology 2014

